# Complete mitochondrial genome of *Dermanyssus gallinae* (Arthropoda: Dermanyssidae) isolate from China

**DOI:** 10.1080/23802359.2021.1923418

**Published:** 2022-01-27

**Authors:** Xiao-Xiao Ma, Yu-Chen Kang, Hu-Dan Li, Qiao-Cheng Chang, Shu-jiang Xue

**Affiliations:** a College of Animal Science and Veterinary Medicine, Heilongjiang Bayi Agricultural University, Daqing, Heilongjiang, P.R. China; b College of Animal Science and Veterinary Medicine, Jilin University, Jilin Province, P.R. China; c School of Public Health, Shantou University, Shantou, Guangdong Province, P.R. China; d Agriculture College of Yanbian University, Yanji, Jilin, P.R. China

**Keywords:** *Dermanyssus gallinae*, mitochondrial genome, phylogenetic analysis

## Abstract

The complete mitochondrial genome of *Dermanyssus gallinae* isolated from China is reported for the first time in this study. Its entire mitogenome is 16, 184 bp in length, contained 13 protein-coding genes, 2 ribosomal RNA genes, 21 transfer RNA genes, and 1 non-coding region. The phylogenetic analysis by maximum likelihood method show that *D. gallinae* isolated from China is in the same clade with the genus of *Psoroptes*. This is the first complete mitochondrial genome of *D. gallinae*.

*Dermanyssus gallinae*, a cosmopolitan hematophagous ectoparasite of birds, is one of the most economically deleterious ectoparasites affecting egg-laying hens worldwide (Cafiero et al. 2019). The infestation burden on caged laying hens can be up to 500,000 mites per bird in severe cases (Kilpinen 2005). In addition to blood loss, as a consequence, the welfare, health and productivity of the birds are severely affected (Cosoroaba 2001). *Dermanyssus gallinae* could serve as a vector for a number of viral and bacterial avian pathogens (Chu et al. 2015). Although several research on *D. gallinae* have done, there is no report on the complete mitochondrial genome. In this study, the complete mitochondrial genome sequence of *D. gallinae* was obtained, which provided a basis for the classification of the genus *Dermanyssus*.

The adult of *D. gallinae* were collected on a chicken farm, Xingtai City, Hebei Province, China (latitude 114.48, longitude 37.07), on 19 June 2019. Individual mite was stored in the Department of Parasitology, Heilongjiang Bayi Agricultural University (qiaocheng Chang; 2001@163.com) under the voucher no. BYNKPL-190619. DNA was extracted from tissues using TruSeq DNA sample Preparation kit. The library with insert size of 400 bp fragments was constructed and sequenced using the Illumina HiSeq platform in Personalbio (Nanjing, China). Illumina paired-end sequencing generated a total of 23,050,688-bp raw reads after removing adapters. A5-miseq and MITOS (Bernt et al. 2013) were utilized for mitogenome assembly and annotation, respectively. The concatenated nucleotide sequences of 13 proteincoding genes were analyzed with maximum likelihood (ML).

The total length of the *D. gallinae* mitochondrial genome was 16,184 bp (GenBank accession no. MW044618), which contained 13 protein-coding genes (*cox*1-3, *nad*1-6, *nad*4L, *atp*6, *atp*8, and *cyt*b), 2 rRNA genes, 21 tRNA genes, and 1 non-coding region. The *D. gallinae* mt genome encoded 3,623 amino acids in total and the concatenated amino acid sequences of 13 protein-coding genes were analyzed with the maximum-likelihood method, using *Anopheles sinensis* (GenBank accession no. NC028016) as the outgroup. The result shows that *D. gallinae* isolated from China is in the same clade with the genus of *Psoroptes* (Figure 1).

**Figure 1.**
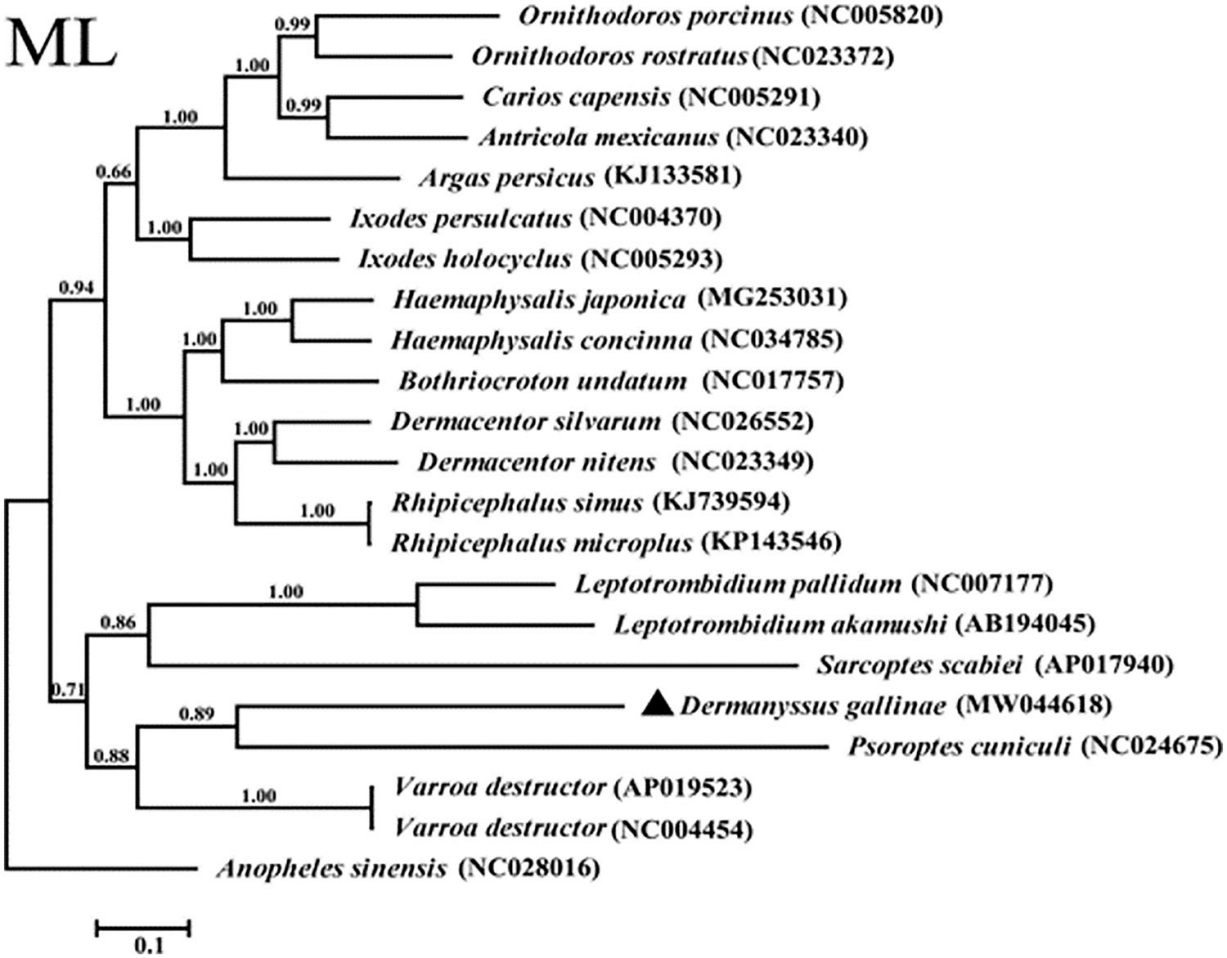
Phylogenetic relationships of *Dermanyssus gallinae* and other species based on mitochondrial sequence data.

## Date availability statement

The genome sequence data that support the findings of this study are openly available in GenBank of NCBI at (https://www.ncbi.nlm.nih.gov) (https://www.ncbi.nlm.nih.gov/) under the accession no. MW044618.
